# OP74 A Comprehensive Health Technology Assessment Framework For Omics Technologies In The Spanish Setting

**DOI:** 10.1017/S0266462324001338

**Published:** 2025-01-07

**Authors:** Patricia Julia Garcia-Sanz, Lorena Aguilera-Cobos, María Piedad Rosario Lozano, Juan Antonio Blasco-Amaro

## Abstract

**Introduction:**

Personalized precision medicine (PPM) is an innovative approach to disease diagnosis, prognosis, and treatment of individual or group characteristics using diverse data sources. While omics technologies are integral to PPM, they pose challenges. Therefore, developing an appropriate methodology to assess these technologies is crucial for patient safety, resource efficiency, and clinical decision-making within the Spanish National Health System.

**Methods:**

This health technology assessment (HTA) methodology procedure was developed by combining three different approaches: a systematic review (SR); a survey targeting experts in omics technology, ranging from basic science researchers to clinicians, and patient associations; and, finally, a consensus method (RAND Appropriateness Method [RAM]).

**Results:**

Through data extraction and evidence synthesis of the 38 studies included in SR, 30 existing frameworks for evaluating omics technologies were identified, as well as the elements needed to assess these technologies, leading to the first version of the framework. Two surveys were performed to integrate the perspectives of omics technology experts and patients. Subsequently, this framework version was further developed by a RAM consensus panel of experts from HTA agencies to ensure a rigorous evaluation of gathered data. The final framework was categorized into 94 elements divided into sections, categories, domains, and subdomains.

**Conclusions:**

A methodological guide, including the assessment framework, was developed for the Spanish HTA network. The framework is divided into several sections addressing evidence-gathering, provision models, organizational elements, economic evaluation, and ethical and social implications. Compared to other available frameworks, our proposal included aspects such as bioinformatics, technological maturity level, and the patient perspective with the personal utility domain.

